# Multiparametric Functional MRI: A Tool to Uncover Subtle Changes following Allogeneic Renal Transplantation

**DOI:** 10.1371/journal.pone.0165532

**Published:** 2016-11-07

**Authors:** Mike Notohamiprodjo, Aivars Kalnins, Martin Andrassy, Manuel Kolb, Benjamin Ehle, Susanna Mueller, Michael N. Thomas, Jens Werner, Markus Guba, Konstantin Nikolaou, Joachim Andrassy

**Affiliations:** 1 Department of Radiology, University Hospital Tuebingen, Tuebingen, Germany; 2 Department of Clinical Radiology, University Hospitals Munich, Munich, Germany; 3 Department of Surgery, University Hospital Munich, Munich, Germany; 4 Department of Medicine, Rupprecht-Karl’s University, Heidelberg, Germany; 5 Department of Pathology, Ludwig-Maximilian’s University, Munich, Germany; University Medical Center Utrecht, NETHERLANDS

## Abstract

**Purpose:**

To investigate multiparametric functional MRI to characterize acute rejection in a murine allogeneic renal transplant model and evaluate the effect of novel therapeutics.

**Material and Methods:**

We performed allogeneic and syngeneic orthotopic transplantations (Balb/c to C57Bl/6 and C57Bl/6 to C57Bl/6). Allogeneic Groups (n = 5) were either treated with the anti-CCL2-Spiegelmer (mNOX-E36) in monotherapy or in combination with low doses of Ciclosporin-A (10mg/kgBW/d) for 10 days. Controls received equivalent doses of a non-functional spiegelmer (revmNOX-E36) or low dose Ciclosporin-A. Diffusion-weighted (DWI) and Dynamic-contrast-enhanced (DCE-) MRI-scans were performed using a clinical 3T-scanner. DWI analysis (b-values from 0–800 s/mm^2^) was performed mono- and biexponentially, while DCE-MRI was assessed with deconvolution analysis. Therapy effects were assessed *ex vivo* with histopathology, immunohistochemistry and RT-PCR. Statistical analysis was performed with unpaired t-tests and Spearman´s correlation coefficient.

**Results:**

DWI showed a significant diffusion restriction in allogeneic compared to syngeneic transplants (ADC: 0.63±0.08 vs. 1.29±0.12 mm^2^/s*10^3^) with decreasing diffusion restriction under therapy. DCE-MRI showed restored organ perfusion under Ciclosporin A alone and combination therapy (Plasma Flow: 43.43±12.49; 38.75±7.53ml/100ml/min) compared to syngeneic controls (51.03±12.49ml/100ml/min). *Ex vivo* analysis showed reduced monocytic infiltrates, attenuated levels of inflammatory cytokines under mNOX-E36 monotherapy with an additive effect of low dose Ciclosporin A. There was a significant (p<0.05) negative correlation between ADC and interstitial inflammation (r = -0.73) or macrophage infiltration (r = -0.81) and between organ perfusion and intimal arteritis (r = -0.63).

**Conclusion:**

Multiparametric functional MRI is suited to detect renal allograft rejection in an experimental murine model and allows to characterize effects of immunosuppressive therapy alleviating acute rejection processes in allogeneic transplantation.

## Introduction

Kidney transplantation renders formidable short term results with one-year graft survival rates of greater than 90% [1]. However, improvements of long term graft survival have been moderate over the course of the last two decades [2]. This holds true not only for renal but for all other solid organ transplants as well [3]. The immunological barriers, the required immunosuppression with their inherent problems and comorbidity of the recipients are the major factors impairing long-term survival [4].

The assessment of treatment effects of immunosuppressive agents as well as the diagnosis of allograft rejection itself is a major problem in transplant medicine. Changes of respective serum levels in combination with loss of function are indirect signs for ongoing allograft injury/rejection. Though biopsies are routinely performed with a limited risk profile, sampling errors and fatal complications with graft loss or even death can occur [5]. Furthermore, histopathology does not necessarily reveal and reflect pathophysiological and functional changes. Functional Magnetic Resonance Imaging (MRI) may serve as an alternative diagnostic non-invasive method [6] Diffusion weighted Imaging (DWI) allows to assess acute allograft rejection by probing molecular water diffusion. Microcirculation can be assessed by an advanced biexponential analysis of the DWI-data applying the intravoxel incoherent motion (IVIM) model or with dynamic contrast enhanced (DCE-)MRI. In exploratory human studies, these non-invasive imaging methods allowed to distinguish between normally functioning organs, acute allograft rejection and ischemic tubular necrosis [7]. However, as of yet, treatment effects have not been evaluated and a direct correlation of MR to histopathological changes has not been performed in the context of renal allograft rejection.

The CCL2-specific l-enantiomeric RNA-Spiegelmer mNOX-E36 neutralizes the biological effects of the murine chemokine MCP1 *in vivo* and *in vitro* and ameliorates leukocyte recruitment and inflammatory response in parenchymal interstitial renal disease [8–10]. Spiegelmers are mirror-image oligonucleotides that are able to bind to a pharmacologically relevant target molecule (in this case the chemokine MCP1) similar to an antibody recognizing an antigen. Due to their specific structure, Spiegelmers cannot be recognized by naturally occurring nucleases, resulting in an increased biostability [11]. In a previous study we demonstrated, that mNOX-E36 has a beneficial effect following murine heart transplantation on the acute rejection process in monotherapy with a strong additive effect in combination with a low dose of Ciclosporin A using manual palpation as clinical reference [12], which is obviously not applicable to renal allografts.

The purpose of this study was to investigate the potential of functional MRI-techniques to non-invasively characterize the acute renal allograft rejection process in a murine allogeneic renal transplant model and evaluate the effect of novel pharmacological therapeutics designed to specifically block CCL2.

## Materials and Methods

### Animals

All procedures involving animals were performed according to the German animal testing Act and approved by the Government of Upper Bavaria (# 55.2-1-54-2531-148-10). C57/Bl6 (H^2b^) and Balb/c (H^2b^) mice (Charles River, Sulzfeld, Germany) were maintained in filter topped cages under standard conditions with free access to a standard diet and water. The animals were 7–14 weeks old at the time of transplantation

### Orthotopic kidney transplantation

A non-life sustaining transplant technique was performed similar as previously described by Russell et al [13]. Briefly, the left kidney was procured. The renal artery and vein were anastomosed to the aorta and inf. V. cava in end-to-side technique. The time from start of the cold perfusion until reperfusion averaged 59.8 ± 10.8 min. The bladder patch was anastomosed to the recipient’s open bladder. The native kidneys of the animals remained in place. Postoperative analgesia was provided with daily buprenorphine s.c.

### Experimental groups

Allogeneic and syngeneic transplantations were performed (Balb/c to C57Bl/6 and C57Bl/6 to C57Bl/6) using 37 donor and 37 recipient mice. Taking into account a perioperative failure rate of 30% (death, urinoma, complete renal infarction) a final group size of n = 5 resulted. The CCL2 binding Spiegelmer **mNOX-E36** (5'-GGCGACAUUGGUUGGG CAUGAGGCGAGGCCCUUUGAUGAAUCCGCGGCCA-3') and the inactive control Spiegelmer **revmNOX-E36** (composed of the reverse nucleotide sequence, both conjugated at their 3´ ends with Y-shaped 40 kDa PEG) (5'-ACCGGCGCCUAAGUAGUUUCCCGGAGCGGA GUACGGGUUG GUUACAGCGG-3') are both modified at the 3'-terminus with 40kDa polyethylene glycol and were synthesized at NOXXON Pharma AG (Berlin, Germany)[14]. The animals received either 15.5 mg (based on oligonucleotide weight)/kg body weight mNOX-E36 or non-functional revmNOX-E36 (serving as a control) intraperitoneally every other day. The Spiegelmer was given either as monotherapy or in combination with a low dose of CsA (10 mg/kgBW/d). One further group of mice received CsA (10 mg/kgBW/d) as monotherapy (**Table 1**). Transplanted animals were monitored via MRI on d10 post transplantation and afterwards sacrificed for further *ex vivo* analysis.

**Table 1:** Treatment Groups.

Group	Therapy	n =
Sygenic (C57Bl/6 to C57Bl/6)	none	5
Allogeneic (Balb/c to C57Bl/6)	Non-functional revmNOX-E36 (15.5mg/kgBW/q.o.d)	5
Allogeneic (Balb/c to C57Bl/6)	Csa (10mg/kgBW/d)	5
Allogeneic (Balb/c to C57Bl/6)	mNOX-E36 (15.5mg/kgBW/q.o.d)	5
Allogeneic (Balb/c to C57Bl/6)	mNOX-36 + CsA	5

### MR Imaging

*In vivo* MR imaging was performed under intraperitoneal Medetomidin-Midazolam-Fentanyl-anesthesia with a clinical 3T-scanner (Magnetom VERIO, Siemens Healthcare Sector, Erlangen, Germany) and a dedicated 8-channel mouse-coil (Rapid Biomedical, Rimpar, Germany) for signal reception. Following morphologic coronal and transversal T1- and T2-weighted sequences, a transversal Echo-Planar-Imaging- sequence (time of repetition = 2600ms, echo time = 90ms) with ten b-values 0, 10, 30, 50, 80, 120, 200, 400, 600 and 800 s/mm^2^ and a resolution of 0.6x0.6x3mm^3^ covering the abdomen was acquired for DWI. For DCE-MRI a TWIST-sequence with a spatial resolution of 0.4x0.4x3mm^3^ and temporal resolution of 1.5 seconds/slab and total acquisition time of 6 minutes was acquired after tail vein injection of 0.05ml/kg Gadobutrol (Gadovist, Bayer Healthcare Pharmaceuticals, Berlin, Germany) in 100μl saline. The paramagnetic Gadolinium-based contrast agent Gadobutrol causes shortening of the T1-time and thus increased signal in T1-weighted sequences, such as the exploited dynamic sequence. Total acquisition time was approximately 30 minutes. The animals were sacrificed after the MRI-examinations by cervical dislocation and exsanguination, while still under anesthesia. The transplanted kidneys were then harvested for further analysis.

### Postprocessing

Postprocessing was performed using the in-house built software PMI 0.4 written in IDL (ITT VIS, Boulder, Colorado, USA). The Apparent Diffusion Coefficient (ADC; mm^2^/s*10^−3^) was derived from whole-kidney ROIs excluding the pelvis defined on parametrical maps calculated from a monoexponential fit of all b-values.

IVIM-metrics for separation of diffusion and pseudodiffusion/perfusion effects were derived as with a voxelwise biexponential analysis [15]. The model for the magnetization M has four parameters: total magnetization M_0_, perfusion fraction f_p_, pseudo-diffusivity D_p_ and tissue diffusivity D_t_:

$$M=M_{0}((f_{p}exp(-bD_{p})+(1–f_{p})exp(-bD_{t}))$$ (1)

A *segmented* IVIM-analysis was performed as described previously [16–19] to ensure a more robust analysis compared to an unconstrained fit, albeit at the expense of some accuracy due to the assumptions involved in the separate measurement of D as follows. When the b-value is significantly greater than 1/D_p_ (e.g. for D_p_ = 10μm^2^/ms, 100s/mm^2^) the pseudodiffusion term is small, so that Eq (1) can be simplified:

$$M_{high}=M_{o}((1-f_{p})exp(-bD_{t}))$$ (2)

D_t_ was determined from a monoexponential fit of the asymptotic high b-values range (b> 200 s/mm^2^). Its zero intercept *M*_*0*_*(1-f*_p_) *= M*_*int*_ is used along with the unweighted (b = 0) signal *M*_*0*_ to determine f_P_.

$$f_{p}=\frac{M_{0}-M_{int}}{M_{0}}$$ (3)

Dp—values were calculated from a biexponential fit with constrained D_t_ and f_p_ according to Eq (1). Parametric maps of the mean D_t_, f_p_, and D_p_ over all directions were generated.

DCE-MRI was analysed based on whole kidney ROIs using model-free deconvolution which is robust and does not impose any constraints on the form of the residue function or the structure of the tissue. It produces a measurement of the impulse response directly from the arterial and tissue tracer concentration. The plasma flow FP (ml/100ml/min) can then be found as the maximum of impulse response. The extracellular volume ECV (ml/100ml) by integration of the impulse response, and the mean transit time (MTT) from the ratio of ECV to FP.

### Reverse transcriptase polymerase chain reaction (RT-PCR)

Total RNA was extracted from kidney samples using Trizol (Invitrogen). To determine the mRNA-expression levels 1 μg total RNA was used to perform reverse transcription and quantitative real time PCR using LightCycler (Roche, Basel, Switzerland) as described previously [20].

Primer sequences were as follows:

**Interferon-g:** forward 5'-TCAAGTGGCATAGATGTGGAAGAA-3' and reverse 5'- TGGCTCTGCAGGATTTTCATG-3'.

**Tumor necrosis factor-α**: forward 5’-CCATTCCTGAGTTCTGCAAG-3', and reverse 5’-GCAAATATAAATAGAGGGGGGC-3'

**B-Cell activating factor:** forward 5’- TCCAGCAGTTTCACAGCGAT-3', and reverse 5’-TTGACTCCAGCGGTCAACTC-3'

**β-Actin**: forward 5'-CCCTAAGGCCAACCGTGAAA-3', and reverse 5'-ACGACCAAGGCATACAGGGA-3'.

Normalization was performed against β-Actin as housekeeping gene.

### Histology

Harvested allografts were split in half and either paraffin embedded or snap frozen and kept at -80°C. Light microscopy was performed on HE- and PAS-stained whole cross sections of kidney allografts. An experienced blinded nephropathologist (S. M.) evaluated and scored interstitial inflammation, intimal arteritis, tubulitis and glomerulitis as well as periarteritis using a 4-point-score (0–3) and assigned a score according to the Banff criteria [21].

### Immunohistochemistry

Immunohistochemistry was performed on 3 μm paraffin embedded cross-sections. Antigen retrieval was performed by Proteinase K (Sigma Aldrich, St. Louis, Missouri) for 20 minutes at 37°C. After blocking with 2.5% goat serum (Vector laboratories, Burlingame, California) the primary antibody (anti–mouse F4/80, 1:100; eBioscience, San Diego, CA) was added for an overnight incubation at 4°C. ImmPRESS HRP kit (Vector laboratories, Burlingame, California) was used for detection. Samples were developed using 3.3'-diaminobenzidine (Sigma Aldrich, St. Louis, Missouri) and nuclear staining was performed using methyl green.

For the analysis five randomly chosen powerfields were analysed per each slide at 20x magnification. Afterwards all slides were analysed using Image J–Image Processing (http://imagej.nih.gov/ij/) adjusting threshold and calculated percents of infiltration area.

### Statistical analysis

Statistical analysis was performed with Microsoft Excel 2013 (Microsoft, Redmond, Washington, USA), SPSS 15 (IBM, Armonk, New York USA) and Prism 6.00 software (GraphPad Software, Inc., San Diego, CA). Differences between groups were investigated with unpaired, two-tailed t-tests after testing for normal variance with the Kolmogorov-Smirnov-method. Correlation between the metric MR parameters and metric leukocyte infiltration was determined with Pearsons´s correleation coefficient and correlation to ordinal histopathology with Spearman´s correlation coefficient. Significance was determined at p<0.05. We have performed Bonferroni-correction for multiple tests.

## Results

### Diffusion Weighted Imaging

**Fig 1:**
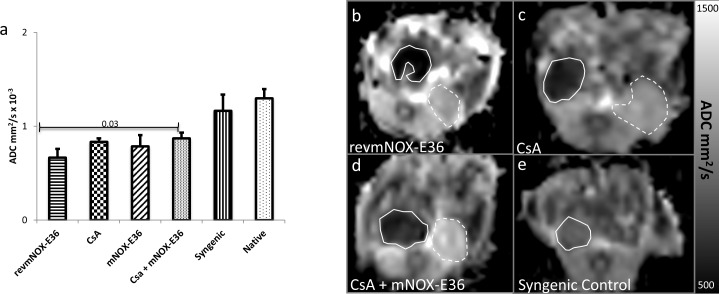
Diffusion Weighted Imaging. (a) Apparent Diffusion Coefficient (ADC): Allograft rejection occurring under revmNOX-E36 shows strong diffusion restriction, i.e. low ADC. Treatment with low dose CsA and mNOX-E36 leads to slight but not significant reduction of the diffusion restriction. Only combined CsA and mNOX lead to a significant increase of ADC, which is still lower than of syngeneic and native kidneys. (b-e) Exemplary parametric ADC-maps (mm^2^/s) of orthotopic renal allografts (continuous lines) and native kidneys (broken lines). (b) shows revmNOX-E36, (c) dose CsA, (d) CsA + mNOX-E36, (e) isograft. The images illustrate a strong diffusion restriction occurring without therapy which partially resolves under low dose CsA and CsA + mNOX-E36. There is only slight diffusion restriction for isografts, attributable to ischemia reperfusion.

The ADC (mm^2^/s*10^−3^) of native kidneys and syngeneic allografts (**Fig 1**) did not show significant differences (1.29±0.12 vs. 1.17±0.16). Allogeneic controls treated with revmNOX-E36 showed significantly lower ADC (0.63±0.08) than syngeneic allografts (p<0.001). Allografts treated with low dose CsA showed a considerably higher ADC (0.78±0.09) (p = 0.05) than revmNOX-E36-controls. Allografts treated with mNOX-E36 only showed a slightly higher ADC (0.83±0.18). The ADC of allografts treated with the combination of CsA and mNOX-E36 were significantly higher (0.90±0.06mm^2^/s) than of revmNOX-E36-controls (p = 0.002) and slightly higher than of mNOX-E36-monotherapy and still significantly lower than of syngeneic kidneys (p<0.001).

IVIM-analysis did not provide additional information (**S1 Fig)** Tissue diffusivity D_t_ of allografts was significantly lower than of sygeneic and native kidneys (p<0.001), however there was no significant difference between the untreated and treated groups. Perfusion fraction f_p_ and pseudodiffusion D_p_ did not show significant differences between all groups.

### Dynamic Contrast Enhanced-MRI

**Fig 2:**
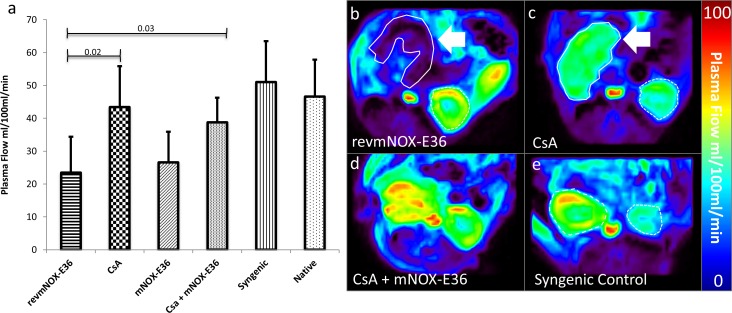
Dynamic Contrast Enhanced–MRI. (a) Plasma flow diagram: Allograft rejection occurring under revmNOX-E36 shows significantly reduced organ perfusion. Treatment with low dose CsA with or without mNOX-E36 leads to significantly increasing organ perfusion, similar to syngeneic and native kidneys. (b-e) Exemplary parametric Plasma flow maps (ml/100ml/min) of orthotopic renal allografts (continuous lines) and native kidneys (broken lines). (b) shows revmNOX-E36, (c) low dose CsA, (d) CsA + mNOX-E36, (e) isograft. The images illustrate a strong reduction of perfusion occurring without therapy. The perfusion deficit resolves already under low dose CsA as well as under CsA + mNOX-E36 with values similar to isografts and native kidneys.

Syngeneic allografts and native kidneys (**Fig 2**) did not show significant perfusion differences (plasma flow (ml/100ml/min): 51.03±12.49 vs. 46.65±11.20) and a significantly higher perfusion than allogeneic allograft controls treated with revmNOX-E36-controls (plasma flow 23.48±10.95). Single treatment with mNOX-E36 did not show significant improvement of perfusion (plasma flow 26.60±9.36). Treatment with low dose CsA without or with mNOX-E36 showed an increasing plasma flow (43.43±12.49; 38.75±7.53 ml/100ml/min) which was significantly higher than in untreated allografts (p<0.01) and mNOX-E36 monotherapy and not significantly different from native kidneys.

### Effect on intragraft proinflammatory cytokines

**Fig 3:**
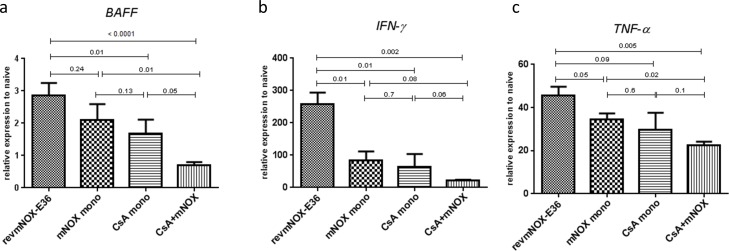
Intragraft pro-inflammatory cytokines. Intragraft mRNA levels of a) B-cell activating Factor (BAFF)-, b) IFN-γ- and c) TNF-α- were significantly reduced under the combination therapy compared to the controls (BAFF: p<0.0001; IFN-γ: p = 0.0002; TNF-α: p = 0.0005). Monotherapy with mNOX-E36 resulted in reduced levels for IFN-γ (p = 0.01 vs. control) and TNF-α (p = 0.05 vs. control) but not for BAFF (p = 0.24). CsA monotherapy significantly reduced IFN-γ- and BAFF-levels (p = 0.01 vs. control) but had less effect on TNF-α (p = 0.09). Combination therapy confirmed again an additive effect over monotherapy.

RT-PCR measurements from the grafts revealed lower levels of B-cell activating Factor (BAFF)-, IFN-γ- and TNF-α- mRNA under the combination therapy compared to revmNOX-E36 treated controls (BAFF:p<0.0001; IFN-γ:p = 0.0002; TNF-α:p = 0.0005) (**Fig 3**). Monotherapy with mNOX-E36 resulted in reduced levels of IFN-γ (p = 0.01 vs. control) and TNF-α (p = 0.05 vs. control). Low dose CsA as monotherapy showed similar results with significantly reduced IFN-γ-levels (p = 0.01 vs. control) and BAFF (p = 0.01 vs. control). Of note, combination therapy confirmed an additive effect over monotherapy especially for BAFF (combination vs. mNOX-E36, p = 0.01; combination vs. CsA, p = 0.05). IFN-γ-concentrations showed a trend (combination vs. mNOX-E36, p = 0.08; combination vs. CsA, p = 0.06). TNF-α-mRNA levels were significantly reduced under the combination compared to mNOX-E36 (p = 0.02) but not to CsA (p = 0.1).

### Histopathology

**Fig 4:**
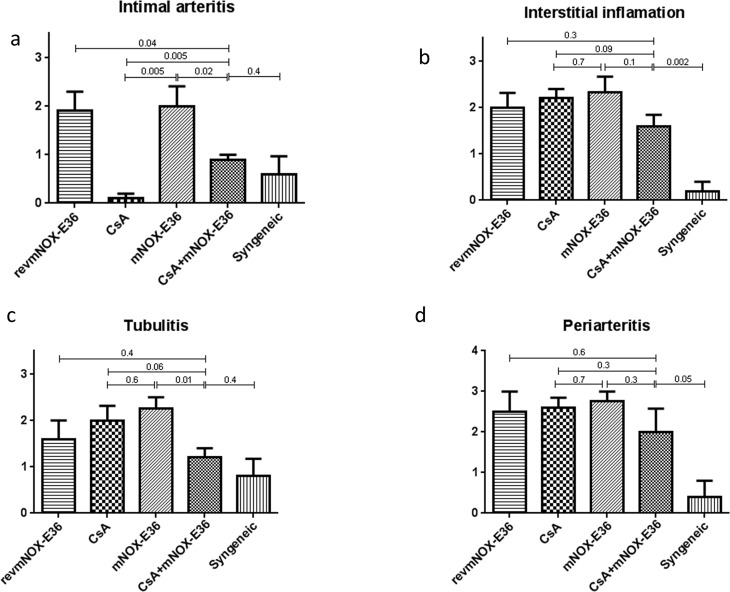
Rejections scores. Rejection scores for a) intimal arteritis, b) interstitial inflammation, c) tubulitis and d) periarteritis were high in all allograft groups. The scores for the combination therapy were significantly reduced only for “intimal arteritis” compared to the controls (p = 0.04). Monotherapy with mNOX-E36 did not show an effect. CsA as monotherapy was significantly reduced only for “intimal arteritis”.

Ten days after transplantation in accordance to the other results, controls (revmNOX-E36) showed most extensive infarction/necrosis (≥ **80%, Fig 4**). In comparison, under mNOX-E36 treatment only 5–10% of the grafts were affected. No infarction was seen under CsA as monotherapy or in combination with mNOX-36.

**Fig 5:**
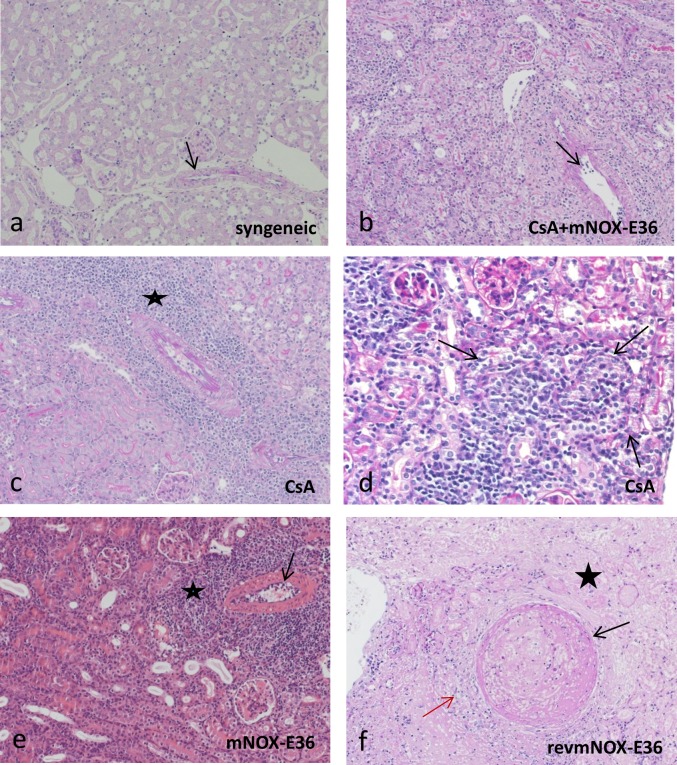
Histopathology of transplant kidneys. magnification 200x; a, b, c, e PAS; d HE a) syngeneic, no therapy (d10 post Tx): no interstitial inflammation, tubulitis or intimal arteritis (arrow); b) allogeneic, CsA+mNOX-E36 (d10 post Tx): mild interstitial inflammation, tubulitis and intimal arteritis (arrow); c) allogeneic, CsA (d10 post Tx): moderate interstitial inflammation and dense perivascular cuff-like lymphocytic inflammation (asterisk), mild intimal arteritis; d) allogeneic, CsA (d10 post Tx): focus of severe tubulitis under CsA monotherapy (arrow); e) allogeneic, mNOX-E36 (d10 post Tx): moderate interstitial inflammation with dense perivascular cuff-like lymphocytic inflammation (asterisk), moderate intimal arteritis (arrow); f) allogeneic, revmNOX-E36 (d10 post Tx): arterial fibrinoid change with medial smooth muscle necrosis (black arrow), mild interstitial inflammation (red arrow). Consecutive ischemic parenchymal necrosis (asterisk).

Syngeneic control renal transplants had almost no signs of inflammation (intimal arteritis 0.6±0.7; interstitial inflammation 0.2±0.4; tubulitis 0.8±0.7; periarteritis 0.4±0.8) and rejection scores were significantly increased in revmNOX-E36 controls (1.9±0.5; 2.0±0.6; 1.6±0.8; 2.5±0.5) and in all treatment groups (interstitial inflammation 1.6 to 2.3; tubulitis 1.2 to 2.3 and periarteritis 2.0 to 2.8) except low dose CsA and combination therapy for intimal arteritis (0.1±0.2 and 0.9±0.2). The combination therapy showed reduced scores compared to either low dose CsA or mNOX-E36 for tubulitis (1.2±4 vs. 2.0±0.6 and 2.3±0.0) and a trend for interstitial inflammation (1.6±0.5 vs 2.2 ± 0.4 and 2.3±0.5) and periarteritis (2.0±1.0 vs. 2.6±0.5 and 2.8±0.4) (**Figs 4 & 5**). According to the Banff-classification these data showed the following picture: no T-cell mediated rejection and minor capillary/glomerular changes for the syngeneic grafts; moderate-severe T-cell mediated rejection for the combination therapy and CsA as monotherapy; severe T-cell mediated changes for mNOX-E36 and revmNOX-E36 (Table 2).

**Table 2:** Banff scores.

	Syngeneic	CsA+mNOX-E36	CsA	mNOX-E36	revmNOX-E36
**Banff-score**	2II, 2II, 2II, 2II, 4IA	3/2II, 4IIA, 4IIA/2II, 4IIA/2II, 4IIA/2II	3/2II, 4IA/2II, 4IA/2II, 4IA/2II, 4IB/2II	4IIB/2II, 4IIB/2II, 4IIB/2II, 4IIB/2II	4IIB/2II, 4IIB/2II, 4IIB/2II, 4III, 4IIB

### Immunohistochemistry

**Fig 6:**
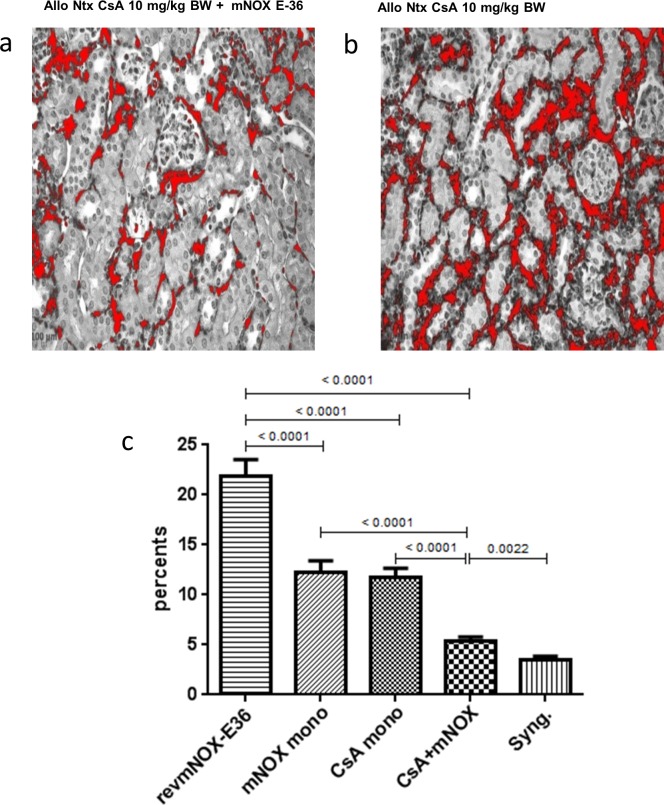
Monocytic infiltrates. Exemplary F4/80+ immunohistochemistry of a specimen treated with (a) low dose CsA + mNOX-E36 or (b) low dose CsA. Under combination therapy considerably less positive F4/80+ cells can be detected. (c) Control grafts (revmNOX-E36) had 22.1±2.3% of the examined area infiltrated with F4/80+ cells. Monotherapy with either mNOX-E36 or low dose CsA showed significant benefits over the controls (10.8±1.8%, p<0.0001). Combination therapy showed an additive effect (5.2±1.2%) significantly reduced compared to both monotherapies and (p<0.0001) almost reaching levels of syngeneic controls (3.4±0.5%).

Immunohistochemical stainings showed that in revmNOX-E36 treated control renal transplants 22.1±2.3% of the examined area was infiltrated with F4/80+ cells (**Fig 6**). This was significantly increased compared to either low dose CsA (10.8±1.5%, p<0.0001) or mNOX-E36 (10.8±1.8%, p<0.0001) as monotherapy. Combination therapy of CsA and mNOX-E36 showed an additive effect (5.2±1.2%), with significantly less monocytic infiltration compared to CsA (p<0.0001) or mNOX-E36 (p<0.0001) monotherapy. Combination therapy reached levels almost comparable to the syngeneic controls (3.4±0.5%).

### Correlation between MRI and histologic parameters

**Fig 7:**
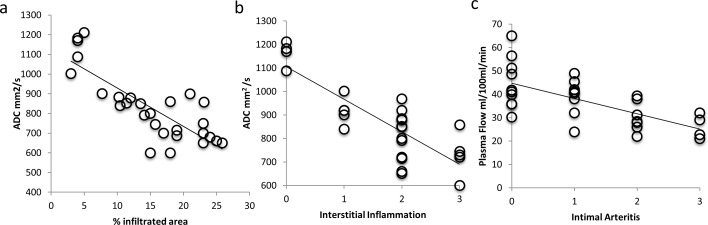
Correlation of functional imaging with histopathology. (a) There was a significant negative correlation between ADC and macrophage infiltration (r = -0.81; p<0.05) determined with immunohistochemistry (F4/80). (b) There was also a significant negative correlation between ADC and interstitial inflammation (r = -0.73; p<0.05) determined with histopatholoy. (c) Furthermore there was a significant negative correlation (r = -0.63; p<0.05) between the reduction of organ perfusion and the degree of intimal arteritis determined with histopathology.

There was a significant (p<0.05) negative correlation (r = -0.63) between perfusion of the transplanted kidney with the degree of the intimal arteritis. Furthermore, we found a significant (p<0.05) negative correlation between interstitial inflammation and ADC (r = -0.73) and between macrophage infiltration and ADC (r = -0.81) (**Fig 7**).

## Discussion

Functional MRI may play a complimentary role to histopathology data in diagnosing parenchymal renal disease and the imaging findings of this study were closely correlated to pathophysiology. This is important as invasive biopsy with all its complications such as haemorrhage or infection is currently the only established method to quantitatively assess renal allograft rejection. While several studies have used functional MRI to depict renal pathology in an animal model [22,23], only two studies have assessed renal allograft rejection using functional MRI [24,25]. Several other studies have used functional MRI to identify transplant rejection in heterogeneous patient cohorts [26–30], but up to date no study has utilized functional MRI to directly assess and graduate therapeutic effects in correlation to histology. In the present study, we exploited functional MRI to detect and grade changes under different treatment regimen and to pinpoint these non-invasive imaging findings to a direct histomorphological correlate.

In allogeneic transplanted controls we could demonstrate considerably impaired mobility of water molecules as measured with DWI, potentially related to a) increased cell density due to MCP1-mediated leukocyte recruitment and b) subsequently increased cell volume due to interstitial inflammation as evidenced by histopathology and RT-PCR [31]. Although more refined diffusion techniques such as diffusion tensor imaging and intravoxel incoherent motion imaging assessing tubular integrity and microcirculation have been developed [27,28,32–34], separation of increased cell density and cell volume is not possible with current techniques. Biexponential analysis yielding the microcirculatory diffusion component based on intravoxel incoherent motion was possible with our acquired data, nevertheless the observed findings were inconsistent with a high degree of variation, potentially due to the complex acquisition and postprocessing technique to separate the flow and diffusion compartments. However, in our study the diffusion restriction assessed with the monoexponential analysis was significantly correlated to leukocyte density and interstitial inflammation and decreased with increasing immunosuppression.

DCE-MRI may help further studying the vascular part of allograft rejection by assessing the organ passage of a contrast agent bolus. We found a significant correlation of perfusion parameters with intimal arteritis and corresponding to histopathology low dose CsA with or without mNOX-E36 led to a reduction of microvascular inflammation, a re-established plasma flow and thus restored microcirculation of the transplanted organ.

Principally, in patients DCE-MRI assessed with multi-compartment-models also allows for calculation of split glomerular filtration if a very high temporal resolution is obtained. Our experiments have been performed with a human scanner using dedicated animal equipment, however an already very high temporal resolution of 1.5sec/slab did not allow for a robust calculation of GFR (data not shown), so that only perfusion data based on a deconvolution analysis was available. Examination with dedicated high field small animal scanners may help to further increase the temporal resolution to non-invasively determine glomerular filtration in mice. Overall we could show that combining DWI with DCE-MRI yielded results closely resembling significant parts of histopathology and immunohistochemistry, particularly leukocyte infiltration and vascular inflammation. However, DWI and DCE-MRI are not the only non-invasive techniques allowing assessment of transplant organs. Arterial Spin Labeling MRI, dynamic contrast enhanced ultrasound (CEUS) and CT (DCE-CT)[35] allow for assessment of perfusion similarly to DCE-MRI and have been used in exploratory studies, particularly addressing ischemia-reperfusion damage. However only DWI allows to non-invasively address tissue cellularity, which is linked to leukocyte recruitment and inflammation [6].

The results regarding effectiveness of the novel chemokine-antagonist mNOX-E36 and its histopathological outcome require further addressing. Only few reports investigated the CCL2/CCR2 axis in the context of organ transplantation, showing that inhibition of the chemokine CCL2 or its receptor CCR2 in the lung and islet allograft rejection process significantly prolongs survival [36,37]. Similar results were obtained in a previous laboratory study of our group using a murine heart transplant model with similar therapy regimen [12]. Here, the combination of mNOX-E36/CsA resulted in reduced monocyte infiltration, interstitial tissue damage and prolonged graft survival. In previously tested parenchymal renal disease models, e.g. diabetes, it was shown that mNOX-E36 effectively blocked macrophage recruitment into the glomerular and interstitial compartments of the kidney [9,10]. Similarly, we found reduced monocytic infiltrates under mNOX-E36 monotherapy. Furthermore, pro-inflammatory cytokines and the ADC as measured by DWI were moderately improved. On the contrary, organ perfusion as measured by DCE-MRI did not recover nor were there improvements using the classical histopathology parameters. When looking at the Banff scores for example, mNOX-E36 was not better than the controls although the extent of infarction/necrosis of the tissue was much less (example Fig 4). Also, the combination therapy failed to show better Banff scores than CsA monotherapy even though the combination led to less interstitial, peritubular and periarterial inflammation. The reason for this was an extraordinary low level of intimal arteritis (v0–0.5) which was even lower than under the combination (v0.5–1). These data were in part confirmed by a non-significant trend towards a better perfusion under CsA mono- vs. combination therapy as measured by DCE-MRI. We lack a good explanation why intimal arteritis was less under CsA monotherapy and can only speculate that the different composition of cellular infiltrates (reduced monocytes under mNOX-E36) or another as of yet unkown cellular/humoral effect is responsible for this.

Importantly, in almost all our experiments a certain additive effect was seen when mNOX-E36 was administered in combination with CsA and that the combination of DCE-MRI and DWI were able to detect these subtle changes.

We furthermore show that histopathology alone with its current scores and classifications lack accuracy. The major weaknesses of the Banff classifications for example are that lesions in the biopsies are empirically derived and thus not specific for any disease entities and their assessment is prone to subjective interpretation and limited reproducibility [38]. These problems have been identified and several Banff working groups are currently focused on a data-driven, evidence-based refinement of the classification [39]. We are convinced that diagnosis of early acute or late ongoing chronic rejection processes should be based on a more multifaceted approach including histology, cellular tests as well as MRI techniques.

### Limitations

The measured ADC values were relatively low compared to values found in human studies, which may be attributed to effects of anesthesia and cooling. However this is a systematic error, which will affect all groups alike. Furthermore group size was small with a final group size of n = 5, however one has to take into account, that the orthotopic kidney transplantation model is very demanding with a perioperative failure rate of approximately 30% [13].

### Conclusion

In summary, multiparametric functional MRI is suited to detect renal allograft rejection in an experimental murine model and allows to characterize effects of immunosuppressive therapy alleviating acute rejection processes in allogeneic transplantation.

## Supporting Information

S1 Fig Intravoxel Incoherent Motion Analysis. (a): Tissue diffusion D_t_: D_t_ is significantly lower for allografts compared to native and syngenic kidneys, however without significant difference between allograft groups. (b) Pseudodiffusion D_p_ and (c) perfusion fraction f_p_ did not show significant differences between all groups.
(TIF)
