# Supplementary figures and images for: Multiparametric Functional MRI: A Tool to Uncover Subtle Changes following Allogeneic Renal Transplantation
Source: PLoS One. 2016 Nov 7;11(11):e0165532. doi: 10.1371/journal.pone.0165532 (PMC5098737; doi:10.1371/journal.pone.0165532)

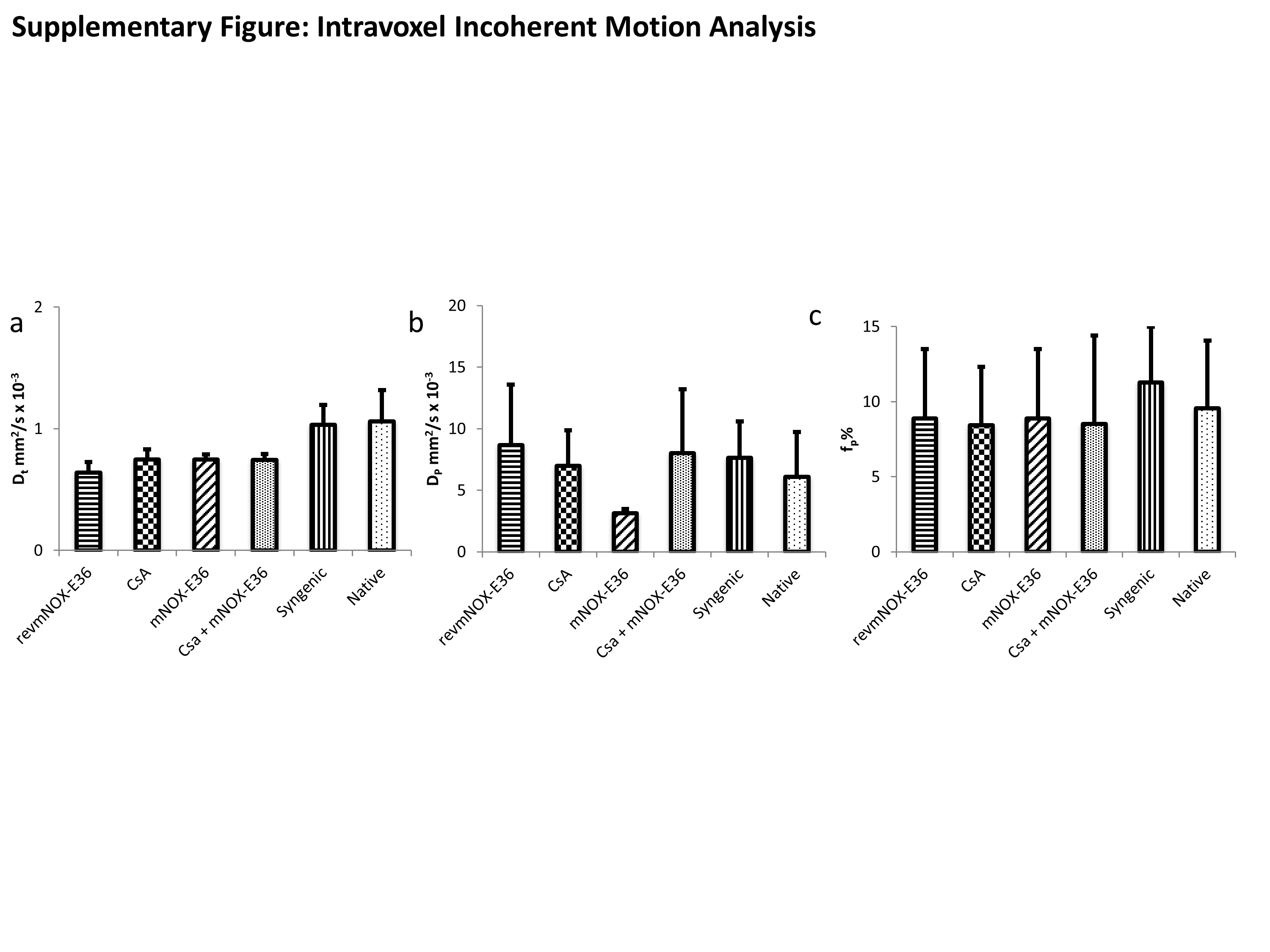

Supplement: S1 Fig — (a): Tissue diffusion Dt: Dt is significantly lower for allografts compared to native and syngenic kidneys, however without significant difference between allograft groups. (b) Pseudodiffusion Dp and (c) perfusion fraction fp did not show significant differences between all groups. (TIF) [file pone.0165532.s001.TIF]
